# Sex difference in the risk of extubation failure in ICUs

**DOI:** 10.1186/s13613-023-01225-7

**Published:** 2023-12-19

**Authors:** Arnaud W. Thille, Florence Boissier, Rémi Coudroy, Sylvain Le Pape, François Arrivé, Laura Marchasson, Jean-Pierre Frat, Stéphanie Ragot, for the REVA Research Network, Grégoire Muller, Arnaud Gacouin, Maxens Decavèle, Romain Sonneville, François Beloncle, Christophe Girault, Laurence Dangers, Alexandre Lautrette, Séverin Cabasson, Anahita Rouzé, Emmanuel Vivier, Anthony Le Meur, Jean-Damien Ricard, Keyvan Razazi, Guillaume Barberet, Christine Lebert, Stephan Ehrmann, Caroline Sabatier, Jeremy Bourenne, Gael Pradel, Pierre Bailly, Nicolas Terzi, Jean Dellamonica, Guillaume Lacave, Pierre-Éric Danin, Hodanou Nanadoumgar, Aude Gibelin, Lassane Zanre, Nicolas Deye

**Affiliations:** 1 Service de Médecine Intensive Réanimation, Centre Hospitalier Universitaire de Poitiers, 2 Rue La Milétrie, 86021 Poitiers Cedex, France; 2 INSERM, CIC 1402 IS-ALIVE, University of Poitiers, Poitiers, France

**Keywords:** Intensive care unit, Airway extubation, Ventilator weaning, Sex difference

## Abstract

**Background:**

Little attention has been paid to potential differences in prognosis between mechanically ventilated males and females in intensive care units (ICUs). We hypothesized that a sex gap in the risk of extubation failure in ICUs may exist.

**Methods:**

Post hoc analysis of a large-scale clinical trial including patients at high risk of extubation failure in ICUs, with the aim of assessing the risk of extubation failure according to sex. The primary outcome was reintubation within the 7 days following extubation.

**Results:**

Out of 641 patients, 425 (66%) were males and 216 (34%) were females. Males were more likely to be admitted for cardiac arrest and to have underlying ischemic heart disease whereas females were more likely to be admitted for coma and to have obesity. Whereas the rate of reintubation at 48 h was significantly higher in males than in females (11.0% vs. 6.0%; difference, + 5.0 [95% CI, 0.2 to 9.2]; *P* = 0.038), the rate of reintubation at day 7 did not significantly differ between males and females (16.7% vs. 11.1%; difference, + 5.6% [95%CI, − 0.3 to 10.8], *P* = 0.059). Using multivariable logistic regression analysis, male sex was independently associated with reintubation within the 7 days following extubation (adjusted OR 1.70 [95% CI, 1.01 to 2.89]; *P* = 0.048), even after adjustment on reason for admission, body-mass index, severity score, respiratory rate before extubation, and noninvasive ventilation after extubation.

**Conclusion:**

In this post hoc analysis of a clinical trial including a homogeneous subset of patients at high risk of extubation failure, sex was independently associated with reintubation. The role of sex on outcomes should be systematically examined in future studies of critically ill patients.

**Supplementary Information:**

The online version contains supplementary material available at 10.1186/s13613-023-01225-7.

## Introduction

In intensive care units (ICUs), the decision of extubation is a critical time insofar as mortality is particularly high in case of reintubation [1–3]. The overall rate of reintubation after planned extubation is around 10% but may exceed 20% in patients at high-risk of extubation failure [1]. Identification of patients at high risk of extubation failure should be considered in order to apply specific measures that may prevent reintubation, such as noninvasive ventilation (NIV) or high-flow nasal oxygen [4, 5]. Many risk factors for extubation failure have been reported in the literature including factors due to underlying comorbidities, acute disease or characteristics at time of extubation [6–8]. The most widely reported risk factors include age over 65 years, history of cardiac disease, chronic respiratory disorders, high severity scores, prolonged duration of mechanical ventilation prior to extubation, ventilatory pattern, hypercapnia at time of extubation, ineffective cough or difficulty to manage secretions [9–15]. Whereas males and females may have different underlying comorbidities, few studies have explored potential differences between males and females in risk of extubation failure. Several studies have reported that the incidence of post-extubation laryngeal edema may be higher in females than in males [16, 17], probably due to an increased ratio of tracheal tube size to laryngeal/tracheal size in females, favoring mechanical injuries [16]. However, aside from this potential difference in risk of post-extubation laryngeal edema, comparison between males and females in ICUs in risk of extubation failure and reintubation remains scarce and underexplored. Females represent less than 40% of mechanically ventilated patients in ICUs [18–21], and therefore, differences between males and females might not be detected in small-scale studies, or even in larger-scale studies that do not make specific adjustments for sex. We believe that potential differences in patient outcomes between males and females mechanically ventilated in ICUs should be carefully examined with proper fit to highlight differences not visible at first glance.

We aimed at assessing the sex gap in the risk of extubation failure from a large-scale clinical trial including patients in ICUs at high risk of reintubation [13]. We hypothesized that the risk of extubation failure leading to reintubation could differ between males and females.

## Methods

### Study design and patients

We performed a post hoc analysis (not pre-specified) of a multicenter (30 centers), randomized, controlled trial comparing NIV alternating with high-flow nasal oxygen versus high-flow nasal oxygen alone within the 48 h following extubation [13]. All patients had been intubated for at least 24 h before extubation and were considered at high risk of extubation failure, i.e., they were older than 65 years or had some underlying chronic cardiac or lung disease [7]. This original study was approved by the central ethics committee (Ethics Committee Ouest III, Poitiers, France) with registration number 2016-A01078-43, and was registered at http://www.clinicaltrials.gov with the trial registration number NCT03121482. Written informed consent was obtained from all patients or next of kin before inclusion.

### Treatment groups

All patients were extubated after successful spontaneous breathing trial performed with a T-piece or pressure-support ventilation according to the physician/center decision (always performed using T-piece in 11 centers, always preformed using pressure-support ventilation in 6 centers, and according to the physician decision in the other 17 centers) [22]. Patients were randomly assigned in a 1:1 ratio (randomization was computer-generated using a centralized web-based management system in permuted blocks of 4 participants) to receive prophylactic NIV alternating with high-flow nasal oxygen or high-flow nasal oxygen alone during the first 48 h following extubation. In the interventional group, NIV was initiated immediately after extubation with minimal duration of at least 12 h a day during the 48 h following extubation. Settings adjusted for NIV and high-flow nasal oxygen are detailed in the original study [13].

### Outcomes

The primary outcome was the proportion of patients who required reintubation within the 7 days following extubation according to sex assigned at birth. Criteria for reintubation were predetermined and patients were immediately reintubated in case of severe respiratory failure if two of the following criteria were fulfilled: respiratory rate > 35 breaths per minute, clinical signs suggesting respiratory distress, respiratory acidosis defined as pH < 7.25 units and PaCO_2_ > 45 mm Hg, hypoxemia defined as PaO_2_/FiO_2_ ≤ 100 mm Hg or FiO_2_ ≥ 80% to maintain SpO_2_ ≥ 92%. The other criteria leading to immediate reintubation included hemodynamic failure with a need for vasopressors, neurological failure (altered consciousness) with a Glasgow Coma Scale < 12, cardiac or respiratory arrest. All criteria and reasons for reintubation were prospectively collected up until ICU discharge. The same patient may have met several criteria or reasons for reintubation**.**

Secondary outcomes included reintubation at 48 h, 72  and up until ICU discharge, post-extubation respiratory failure within the 7 days following extubation, length of stay in ICU and in hospital, and mortality in ICU and in hospital and up to 90 days after extubation according to sex. Post-extubation respiratory failure was defined by the presence of at least two criteria among the following: respiratory rate > 25 breaths per minute, clinical signs suggesting respiratory distress, respiratory acidosis defined as pH < 7.35 units and PaCO_2_ > 45 mm Hg, and hypoxemia defined as PaO_2_/FiO_2_ ≤ 150 mm Hg or FiO_2_ ≥ 50% to maintain SpO_2_ ≥ 92%. We asked the investigators to specify whether the main reason for post-extubation respiratory failure was due to cardiogenic pulmonary edema or post-extubation laryngeal edema.

### Statistical analysis

All of the analyses were performed by the study statistician (S.R.). Continuous variables were expressed as mean ± standard deviation or median [interquartile range, 25th–75th percentiles], and qualitative variables as number and percentage.

Males and females were compared by means of the χ^2^ tests or Fisher exact test for categorical variables and Student’s *t* test or Wilcoxon test for continuous variables as appropriate.

Kaplan–Meier curves were plotted in males and females to assess the probability of reintubation during the 7 days following extubation, censoring at the date of reintubation, death or at day 7. Patients discharged from ICU before day 7 were followed for reintubation until day 7 (primary outcome), and therefore, were not censored on the day of ICU discharge. A log-rank test was used to compare the cumulative incidence of reintubation within the 7 days following extubation between males and females.

A multivariable logistic regression analysis was performed for the primary outcome. Variables associated with reintubation within the 7 days following extubation with a p value of less than 0.10 using univariable analysis were entered into the maximal model. Regarding the association between sex and reintubation, an interaction test was performed between sex and each of all other covariates included in the maximal model. Normality of all the quantitative variables was checked using a quantile–quantile (Q–Q) plot and a Shapiro–Wilk test for normality. Multicollinearity of highly correlated predictor variables was assessed using variance inflation factors. We also performed a Cox model for the primary outcome as a sensitivity analysis. A two-tailed p value of less than 0.05 was considered statistically significant. We used SAS software, version 9.4 (SAS Institute), for all the analyses.

## Results

Out of 641 patients at high risk of extubation failure included in the original study, 425 (66%) were males and 216 (34%) were females. Males were more likely to be admitted for cardiac arrest and to have underlying ischemic heart disease whereas females were more likely to be admitted for coma and to have obesity and obesity-hypoventilation syndrome (Table 1). Before extubation, females had lower tidal volume, higher respiratory rate and higher rapid shallow breathing index (F/V_T_) despite higher levels of pressure-support level and PEEP, and were less likely to have abundant secretions. Females tended to have a lower severity score as indicated by lower Sequential Organ Failure Assessment (SOFA) score at the time of extubation than males but it was not significant. After extubation, the proportion of patients receiving NIV did not significantly differ between males and females. Within the first 24 h following extubation, NIV was applied for 14.0 [IQR, 11.8–15.9] hours in males and 13.6 [IQR, 11.1–16.0] hours in females (*P* = 0.490).

**Table 1:** Comparison of patient characteristics between males and females

	Male (*n* = 425)	Female (*n* = 216)	*P* value*
Characteristics of the patients at admission			0.381
Age, years	69 ± 10	70 ± 11	0.164
Age > 65 years, *n* (%)	298 (70%)	162 (75%)	0.194
Body-mass index, kg/m^2^	28 ± 6	29 ± 8	**0.001**
Body-mass index ≥ 30 kg/m^2^, *n* (%)	121 (29%)	85 (41%)	**0.004**
SAPS II at admission, points	55 ± 18	56 ± 19	0.494
Underlying chronic cardiac disease, *n* (%)	218 (51)	88 (41%)	**0.011**
Ischemic heart disease	129 (30%)	37 (17%)	** < 0.001**
Atrial fibrillation	66 (15%)	37 (17%)	0.602
Left ventricular dysfunction	61 (14%)	30 (14%)	0.873
History of cardiogenic pulmonary edema	31 (7%)	15 (7%)	0.871
Underlying chronic lung disease, *n* (%)	143 (34%)	70 (32%)	0.752
Chronic obstructive pulmonary disease	109 (26%)	41 (19%)	0.059
Obesity-hypoventilation syndrome	25 (6%)	24 (11%)	**0.018**
Chronic restrictive pulmonary disease	25 (6%)	11 (5%)	0.681
Main reason for admission, No. (%)			**0.026**
Acute respiratory failure	202 (47%)	113 (52%)	0.251
Coma	41 (10%)	34 (16%)	**0.023**
Shock	48 (11%)	15 (7%)	0.080
Cardiac arrest	48 (11%)	13 (6%)	**0.031**
Surgery	41 (10%)	22 (10%)	0.828
Other reason	45 (11%)	19 (9%)	0.474
Characteristics of the patients on the day of extubation			
SOFA score, points	4.4 ± 2.7	4.0 ± 2.5	0.051
Duration of mechanical ventilation, days	6 [3–10]	5 [3–10]	0.901
Difficult or prolonged weaning #, *n* (%)	133 (31%)	73 (34%)	0.521
Ineffective cough, n/n total (%)	95/400 (24%)	56/206 (27%)	0.354
Abundant secretions, n/n total (%)	170/406 (42%)	65/208 (31%)	**0.010**
Administration of steroids before extubation, *n* (%)	66 (15%)	29 (13%)	0.478
Ventilatory settings before the spontaneous breathing trial			
Pressure-support ventilation, *n* (%)	369 (87%)	179 (83%)	0.197
Pressure-support level, cm H_2_O	9.3 ± 2.7	9.9 ± 3.1	**0.019**
Positive end-expiratory pressure, cm H_2_O	5.7 ± 1.6	6.0 ± 1.5	**0.042**
Respiratory rate, breaths/min	22 ± 6	23 ± 7	**0.007**
Tidal volume, ml	505 ± 140	413 ± 102	**< 0.001**
Tidal volume, ml/kg of predicted body weight	7.5 ± 2.2	8.1 ± 2.3	**0.005**
Rapid shallow breathing index: F/V_T_, breaths/min/l	48 ± 26	60 ± 26	**< 0.001**
FiO_2_, %	35 ± 11	35 ± 10	0.949
PaO_2_/FiO_2_, mm Hg	277 ± 91	268 ± 90	0.215
pH, units	7.45 ± 0.06	7.45 ± 0.06	0.501
PaCO_2_, mm Hg	39 ± 7	40 ± 9	0.153
PaCO_2_ > 45 mm Hg, No. (%)	78 (18%)	44 (20%)	0.467
Characteristics of the spontaneous breathing trial			
Type of trial			
T-piece trial, *n* (%)	262 (62%)	132 (61%)	0.895
Low level of pressure-support, *n* (%)	163 (38%)	84 (39%)	
Median duration of the trial, min	60 [30–60]	60 [30–60]	0.916
Respiratory support after extubation			
Prophylactic noninvasive ventilation, *n* (%)	230 (54%)	109 (50%)	0.381
High-flow nasal oxygen, *n* (%)	195 (46%)	107 (50%)	

Continuous variables are given in mean ± standard deviation or median [interquartile range, IQR 25th – 75th percentiles] according to their distribution

^*^ In bold all variables significantly different between males and females with a p value of less than 0.05 are indicated

**Abbreviations:** SAPS = Simplified Acute Physiology Score; SOFA = Sepsis-related Organ Failure Assessment; SBT = Spontaneous Breathing Trial; F/V_T_ = rapid shallow breathing index calculated as the ratio of respiratory frequency (F) divided by tidal volume (V_T_)

^#^ Difficult or prolonged weaning refers to patient who failed the first spontaneous breathing trial and were not extubated the day of the first trial

### Comparison of outcomes between males and females

**Fig. 1:**
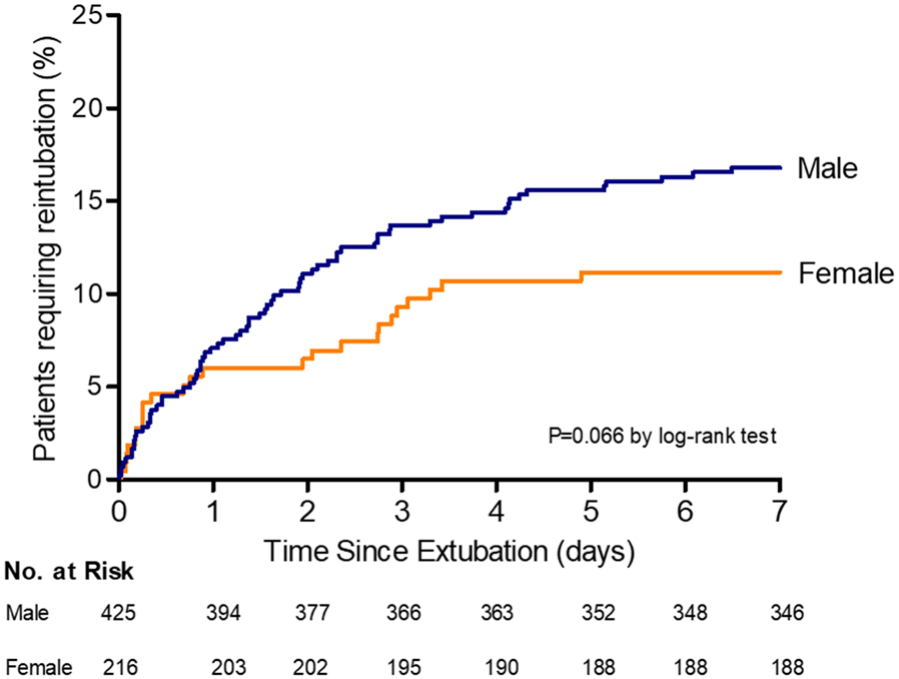
Kaplan–Meier analysis of time from extubation to reintubation according to sex. The rate of reintubation within the 7 days following extubation was 16.7% in males (blue line) and 11.1% in females (orange line); *P* = 0.066 using log-rank test

Whereas the rate of reintubation within the first 48 h following extubation was significantly higher in males than in females (11.0% vs. 6.0%; difference, + 5.0 [0.2 to 9.2]; *P* = 0.038), the rate of reintubation within the 7 days following extubation rate did not significantly differ between males and females (16.7% vs. 11.1%; difference, + 5.6% [95% CI, − 0.3 to 10.8], *P* = 0.059) (Table 2) (Fig. 1).

**Table 2:** Comparison of outcomes between males and females

	Male (*n* = 425)	Female (*n* = 216)	Absolute difference % (95% CI)	*P* value*
Primary outcome				
Reintubation at day 7, *n* (%)	71 (16.7%)	24 (11.1%)	5.6 (− 0.3 to 10.8)	0.059
Secondary outcomes				
Post-extubation respiratory failure at day 7, *n* (%)	103 (24.2%)	55 (25.4%)	− 1.2 (− 8.5 to 5.6)	0.733
Cardiogenic pulmonary edema, *n* (%)	19 (4.5%)	8 (3.7%)	0.8 (− 3.0 to 3.8)	0.648
Upper airway obstruction, *n* (%)	15 (3.5%)	12 (5.5%)	− 2.0 (− 6.2 to 1.2)	0.227
Reintubation among patients with post-extubation respiratory failure, n/n total (%)	57/103 (55.3%)	22/55 (40.0%)	15.3 (− 1 to 30.4)	0.066
Reintubation at 48 h, *n* (%)	47 (11.0%)	13 (6.0%)	5.0 (0.2 to 9.2)	**0.038**
Reintubation at 72 h, *n* (%)	58 (13.6%)	19 (8.7%)	4.9 (− 0.6 to 9.6)	0.074
Reintubation in ICU, *n* (%)	74 (17.4%)	26 (12.0%)	5.4 (− 0.6 to 10.7)	0.076
Patient meeting reintubation criteria in ICU, *n* (%)	84 (19.8%)	30 (13.9%)	5.9 (− `0.4 to 11.6)	0.066
Length of stay in ICU, days	12 [7–18]	10 [6–16]	–	0.070
Length of stay in hospital, days	16 [9–32]	15 [8–24]	–	0.091
Mortality in ICU, *n* (%)	32 (7.5%)	15 (6.9%)	0.6 (− 4.1 to 4.6)	0.782
Mortality in hospital, *n* (%)	73 (17.2%)	27 (12.5%)	4.7 (− 1.4 to 10.1)	0.123
Mortality at day-90, *n* (%)	93 (21.9%)	35 (16.2%)	5.7 (− 0.9 to 11.7)	0.089

Lengths of stay are given in median [interquartile range, IQR 25th – 75th percentiles]

^*^ In bold are indicated all variables significantly different between males and females with a p value of less than 0.05

The proportion of patients who experienced post-extubation respiratory failure did not significantly differ between males and females, nor did the proportion of patients with post-extubation respiratory failure due to cardiogenic pulmonary edema or upper airway obstruction. Among patients with post-extubation respiratory failure, males tended to be more frequently intubated than females but the difference was not significant. (55.3% vs. 40.0%; difference, + 15.3% [95% CI, − 1 to 30.4], *P* = 0.066).

Criteria and reasons for reintubation did not significantly differ between males and females (Additional file 1: Table S1).

Length of stay in ICU and in hospital, and mortality up until 90 days after inclusion did not significantly differ between males and females (Table 2).

### Variables associated with reintubation within the 7 days following extubation

Patients successfully extubated had been more frequently admitted for cardiac arrest and had higher body-mass index than those patients who required reintubation (Table 3). Patients who required reintubation had a higher SOFA severity score, and higher pressure-support level before extubation than successfully extubated patients. Use of prophylactic NIV after extubation was associated with a significant decreased risk of reintubation. Males and patients with a high respiratory rate before extubation tended to have an increased risk of reintubation but it was not significant (*P* < 0.10).

**Table 3:** Comparison between patients who required reintubation within the 7 days following extubation and those who were successfully extubated

	Extubation success (*N* = 546)	Reintubation at day 7 (*N* = 95)	*P* value*
Characteristics of the patients at admission			
Sex male, *n* (%)	354 (65%)	71 (75%)	**0.059**
Age, years	70 ± 10	70 ± 8	0.599
Age > 65 years, *n* (%)	389 (71%)	71 (75%)	0.485
Body-mass index, kg/m^2^	28 ± 7	27 ± 6	**0.026**
Body-mass index ≥ 30 kg/m^2^, *n* (%)	182 (34%)	24 (26%)	0.107
SAPS II at admission, points	55 ± 19	57 ± 18	0.381
Underlying chronic cardiac disease, *n* (%)	266 (49%)	40 (42%)	0.233
Ischemic heart disease	143 (26%)	23 (24%)	0.684
Atrial fibrillation	90 (16%)	13 (14%)	0.492
Left ventricular dysfunction	77 (14%)	14 (15%)	0.870
History of cardiogenic pulmonary edema	39 (7%)	7 (7%)	0.937
Underlying chronic lung disease, *n* (%)	181 (33)	32 (34)	0.918
Chronic obstructive pulmonary disease	122 (22%)	28 (29%)	0.129
Obesity-hypoventilation syndrome	46 (8%)	3 (3%)	**0.074**
Chronic restrictive pulmonary disease	32 (6%)	4 (4%)	0.519
Main reason for admission			**0.042**
Acute respiratory failure	273 (50%)	52 (55%)	0.394
Coma	96 (18%)	16 (17%)	0.860
Shock	56 (10%)	11 (12%)	0.697
Cardiac arrest	59 (11%)	2 (2%)	**0.007**
Surgery	53 (10%)	10 (11%)	0.817
Other reason	9 (2%)	4 (4%)	0.111
Characteristics of the patients on the day of extubation			
SOFA score, points	4.2 ± 2.6	4.8 ± 2.8	**0.027**
Duration of mechanical ventilation, days	5 [3–10]	6 [3–10]	0.316
Difficult or prolonged weaning #, *n* (%)	176 (32%)	30 (32%)	0.970
Ineffective cough, *n*/*n* total (%)	123 (24%)	28 (30%)	0.184
Abundant secretions, *n*/*n* total (%)	194 (37%)	41 (45%)	0.178
Administration of steroids before extubation, *n* (%)	86 (16%)	9 (9%)	0.112
Ventilator settings before the spontaneous breathing trial			
Pressure-support ventilation, *n* (%)	467 (86%)	81 (85%)	0.945
Pressure-support level, cm H_2_O	9 ± 3	10 ± 3	**0.049**
Positive end-expiratory pressure, cm H_2_O	6 ± 2	6 ± 2	0.173
Respiratory rate, breaths/min	22 ± 6	23 ± 7	**0.089**
Tidal volume, ml	474 ± 136	475 ± 129	0.953
Tidal volume, ml/kg of predicted body weight	7.8 ± 2.2	7. 5 ± 2.2	0.319
Rapid shallow breathing index: F/V_T_, breaths/min/l	51 ± 23	55 ± 30	0.194
FiO_2_, %	35 ± 10	35 ± 12	0.695
PaO_2_/FiO_2_, mm Hg	275 ± 91	268 ± 91	0.446
pH, units	7.45 ± 0.06	7.45 ± 0.06	0.999
PaCO_2_, mm Hg	40 ± 8	39 ± 8	0.567
PaCO_2_ > 45 mm Hg, No. (%)	101 (19%)	21 (22%)	0.443
Characteristics at the end of the spontaneous breathing trial			
Type of trial			
T-piece trial, *n* (%)	336 (62%)	58 (61%)	0.928
Low level of pressure-support, *n* (%)	210 (38%)	37 (39%)	
Median duration of the trial, min	60 [30–60]	45 [30–60]	0.159
Respiratory support after extubation			
Prophylactic noninvasive ventilation, *n* (%)	299 (55%)	40 (42%)	**0.023**
High-flow nasal oxygen, *n* (%)	247 (45%)	55 (58%)	

Continuous variables are given in mean ± standard deviation or median [interquartile range, IQR 25th–75th percentiles] according to their distribution

*In bold are indicated all variables associated with reintubation within the 7 days following extubation with a *P* value of less than 0.10 using univariable analysis

^#^Difficult or prolonged weaning refers to patient who failed the first spontaneous breathing trial and were not extubated the day of the first trial

SAPS = Simplified Acute Physiology Score; SOFA = Sequential Organ Failure Assessment; SBT = Spontaneous Breathing Trial; F/V_T_ = rapid shallow breathing index calculated as the ratio of respiratory frequency (F) divided by tidal volume (V_T_)

### Multivariate logistic regression and Cox model

Using multivariable logistic regression, higher body-mass index, admission for cardiac arrest and prophylactic NIV after extubation were independently associated with successful extubation, whereas higher SOFA severity score, higher respiratory rate before extubation and male sex were independently associated with reintubation within the 7 days following extubation (Table 4).

**Table 4:** Multivariate logistic regression analysis of factors associated with reintubation within the 7 days following extubation

Variables independently associated with reintubation	Adjusted odds ratio [95% confidence interval]	*P* value
Variables associated with an increased risk of reintubation		
Male sex (women as reference)	1.70 [1.01–2.89]	**0.048**
SOFA score the day of extubation—for each point increase	1.09 [1.01–1.19]	**0.034**
Respiratory rate before extubation—for each breath increase	1.04 [1.00–1.07]	**0.048**
Variables associated with a decreased risk of reintubation		
Body-mass index—for each point increase	0.96 [0.92–0.99]	**0.039**
Cardiac arrest as main reason of admission	0.17 [0.04–0.70]	**0.014**
Use of prophylactic noninvasive ventilation after extubation	0.56 [0.35–0.89]	**0.015**

SOFA = Sequential Organ Failure Assessment

*In bold are indicated all variables independently associated with reintubation within 7 days following extubation in the final model using multivariable analysis with a p value of less than 0.05

All variables associated with reintubation with a *P* value < 0.10 were included in the model (R^2^ = 0.06): (1) Male sex, (2) SOFA score the day of extubation, (3) Respiratory rate before extubation, (4) Body-mass index, (5) Cardiac arrest as main reason of admission, (6) Use of prophylactic noninvasive ventilation after extubation

Male sex was independently associated with reintubation within the 7 days following extubation (OR 1.70 [95% CI, 1.01–2.89]; *P* = 0.048), even after adjustment on body-mass index, admission for cardiac arrest, SOFA severity score and respiratory rate before extubation, and use of prophylactic noninvasive ventilation after extubation. There was no interaction between sex and reintubation. No multicollinearity was found between all independent variables and estimates of regression coefficients were stable. Using Cox model, sex remained independently associated with cumulative incidence of reintubation within the 7 days following extubation with an adjusted hazard ratio of 1.66 [95% CI, 1.02–2.70]; *P* = 0.042.

## Discussion

In this post hoc analysis of a clinical trial including a homogeneous subset of patients at high risk of extubation failure in ICUs, sex was associated with a significant difference in the risk of reintubation. Whereas males were less likely to have obesity than females, they were more likely to have underlying ischemic heart disease, and were more frequently admitted to the ICU for cardiac arrest. The rate of reintubation within the 48 h following extubation was significantly higher in males than in females. After multivariable logistic regression analysis, males had an increased risk of reintubation within the 7 days following extubation, regardless of the reason for admission, body-mass index, severity score, respiratory rate before extubation, and noninvasive respiratory support used after extubation.

### Sex differences in ICUs

We report here that female sex was independently associated with decreased risk of reintubation. Females are largely in the minority in ICUs, representing less than 40% of admissions [18–21]. The reasons for their less frequent admission to ICUs have been poorly explored, but could be explained by a difference in the prevalence of underlying cardiac/respiratory disorders or other comorbidities, or by inherent sex-related difference [21]. Few studies have compared outcomes between males and females admitted to ICUs, with substantial heterogeneity among studies and risk of bias [21, 23], and it is likely that potential differences in outcomes in ICUs have been under-examined. The few studies having evaluated the impact of sex on the outcome of mechanically ventilated patients present with discordant results [24–27].

In a previous study including 225 patients extubated after successful spontaneous breathing trial, we had already found a trend toward a decreased risk of reintubation in females (the rate of reintubation at day 7 was 8.7% (8/92) in females and 17.3% (23/133) in males; *P* = 0.077) [8]. Although sex was not significantly associated with reintubation after multivariable logistic regression analysis, this study may have been underpowered to detect a significant difference. It is also possible that regarding outcomes in ICUs, the sex variable has not been assessed with the full attention that it warrants. Statistical models are built by statisticians and clinicians, and the role of sex in outcomes may be insufficiently covered. In a recent systematic review of more than 22,000 patients assessing all risk factors of extubation failure, sex was not associated with reintubation [6]. However, this systematic review included all studies assessing risk factors of extubation failure, i.e., studies including patients at low risk of extubation failure such as young people, postoperative patients or those with short duration of mechanical ventilation without any underlying comorbidities. In the present study, we included only patients at high risk of extubation failure, and differences between males and females may appear in older patients, who are more likely to have underlying cardiac or chronic lung disease.

The reasons for a sex difference in the risk of extubation failure in ICUs remain unclear. However, males were more likely to have underlying ischemic heart disease than females and history of cardiac disease or heart failure are well-established and consensual risk factors for reintubation [5–8, 10]. By contrast, females were more likely to have obesity than males and this could contribute to prevent extubation failure. Although obesity has previously been considered as a means of identifying patients at high risk of extubation failure [14, 15], a systematic review assessing all factors associated with reintubation in ICUs showed that on the contrary, obesity was associated with a decreased risk of extubation failure [6]. In keeping with this study, we found that the higher the body-mass index, the lower the risk of reintubation. This could be explained by the fact that patients with obesity are particularly good responders to noninvasive ventilation, with a rate of reintubation particularly low once under positive pressure [28, 29]. A recent study including only patients at high risk of extubation failure, i.e., older than 65 years or with underlying cardiac or chronic lung disease, reported a rate of reintubation among the 112 obese patients receiving noninvasive ventilation of only 6% within the 7 days following extubation, and only 2% at 48 h [28].

We also found that the F/V_T_ ratio before extubation was subsequently markedly higher in females than in males. Respiratory rate was higher and tidal volumes were lower in females than in males, and we cannot rule out that a high F/V_T_ ratio may have unduly delayed extubation and may have decreased the risk of post-extubation respiratory failure in females. Although the F/V_T_ ratio has been proposed as a good predictor of successful weaning [30], a previous study showed that females had higher F/V_T_ ratio than males, independent of extubation outcome [31]. In our study, females had lower tidal volumes than males when expressed in milliliters but higher tidal volumes than males when expressed in milliliters per kilograms of predicted body weight taking into account the sex and the height. This could explain that the F/V_T_ ratio is not a reliable predictor of extubation outcome and could even delay extubation, especially in females [32]. In a Canadian registry including 3743 patients, it was shown that extubation was more frequently withheld in females than in males despite successful spontaneous breathing trial [33]. Although this was not the case in this registry, delayed extubation may have influenced extubation outcome.

Another hypothesis may be that a pro-inflammatory sex hormone profile may be associated with acute respiratory distress syndrome as previously suggested [21], and that respiratory disease severity may then impact weaning period and extubation outcome.

Lastly, delirium could be more frequent in males than in females [34], and one can hypothesize that this acute brain dysfunction during the weaning period may also precipitate extubation failure.

### Risk factors of extubation failure

Outside of sex, we found that reason for admission, body-mass index, severity score, respiratory rate before extubation, and noninvasive respiratory support after extubation were independently associated with success or failure of extubation. Cardiac arrest as a reason for admission had not previously been considered in terms of risk of extubation failure, and it should be explored in future studies. Indeed, cardiac arrest with acute and transient heart failure could be less harmful in terms of risk of extubation failure than chronic heart failure. As above-emphasized and contrary to what one might suppose, body-mass index was associated with decreased risk of reintubation. Respiratory pattern, especially high respiratory rate before extubation, as well as high severity score at the time of extubation, are well-known risk factors for reintubation, even though they are expressed as continuous variables and no precise threshold can constructively contribute to the decision to extubate [6]. Lastly, several clinical trials have shown a decreased risk of reintubation with prophylactic noninvasive ventilation in patients at high risk of extubation failure [9, 13, 35], and this noninvasive respiratory support is currently recommended in this setting [4, 5].

### Limitations

The main limitation is that this post hoc analysis was not pre-planned. Although our result is only an exploratory analysis, we believe that sex differences should be systematically examined in futures prospective studies, especially during the weaning and post-extubation period.

Several studies have reported that the incidence of post-extubation laryngeal edema was higher in females than in males, which is probably explained by an increased ratio of tracheal tube size to laryngeal/tracheal size in females, a factor favoring mechanical injuries [16, 17]. Another study has even suggested that females may be at increased risk of reintubation due to airway failure within the first 48 h following extubation [36]. However, as defined in this study airway failure included not only upper airway obstruction, but also other reasons for reintubation such as aspiration, ineffective cough or inability to clear abundant secretions. Even though post-extubation laryngeal edema may occur more frequently in females than in males, the above-mentioned studies did not report any gap in the risk of extubation failure due to upper airway obstruction [16, 17, 36]. Although we made a point of prospectively collecting all reasons for reintubation, the proportion of patients reintubated for upper airway obstruction due to post-extubation laryngeal edema was uncommon and did not differ between males and females.

To our knowledge, no previous studies have mentioned a difference between males and females regarding management of ventilator weaning or airway extubation. One cannot rule out that the role of sex in patient outcomes in ICUs has been insufficiently explored. Although this first study alone does not make it possible to affirm with certainty a difference in the risk of extubation failure between males and females in ICUs, we believe a sex gap should be systematically examined in the future.

## Conclusion

In this post hoc analysis of a clinical trial including patients at high risk of extubation failure, males were at an increased risk of reintubation compared to females after adjustment on reason for admission, body-mass index, severity score before extubation, respiratory rate before extubation, and noninvasive respiratory support used after extubation. Sex gap has been poorly explored in ICUs, and thereby, the role of sex on outcomes should be systematically examined in future studies of critically ill patients.

### Supplementary Information

**Additional file 1.** **Table S1:** Comparison of criteria and reasons for reintubation between males and females.

## Data Availability

The datasets used and/or analyzed during the current study are available from the corresponding author on reasonable request.
