# Supplementary material for: Sex difference in the risk of extubation failure in ICUs
Source: Ann Intensive Care. 2023 Dec 19;13:130. doi: 10.1186/s13613-023-01225-7 (PMC10730492; doi:10.1186/s13613-023-01225-7)
Supplement: Supplementary file 1 — Additional file 1. Table S1: Comparison of criteria and reasons for reintubation between males and females. [file 13613_2023_1225_MOESM1_ESM.docx]

**Table S1: Comparison of criteria and reasons for reintubation between males and females.**

|  | **Male (n=425)** | **Female (n=216)** | **P value** |
| --- | --- | --- | --- |
| ***Reintubation up until ICU discharge, n (%)*** | 74 (17%) | 26 (12%) | 0.076 |
| ***Criteria for reintubation*********, n (%)*** |  |  |  |
| - Severe respiratory failure, n (%) | 65 (15%) | 23 (11%) | 0.106 |
| - Neurological failure, n (%) | 25 (6%) | 12 (6%) | 0.867 |
| - Hemodynamic failure, n (%) | 11 (3%) | 5 (2%) | 0.834 |
| - Respiratory or cardiac arrest, n (%) | 8 (2%) | 2 (1%) | 0.356 |
| ***Reasons for reintubation*********, n (%)*** |  |  |  |
| Cardiogenic pulmonary edema, n (%) | 11 (3%) | 5 (2%) | 0.834 |
| Upper airway obstruction, n (%) | 6 (1%) | 6 (3%) | 0.233 |
| Aspiration, n (%) | 2 (0%) | 1 (0%) | 0.999 |
| Pneumonia, n (%) | 8 (2%) | 2 (1%) | 0.508 |
| Atelectasis, n (%) | 7 (2%) | 6 (3%) | 0.379 |
| Pleural effusion, n (%) | 3 (1%) | 2 (1%) | 0.999 |
| Pneumothorax, n (%) | 2 (0%) | 1 (0%) | 0.999 |
| Inability to clear secretions, n (%) | 29 (7%) | 7 (3%) | 0.063 |
| Ineffective cough, n (%) | 16 (4%) | 5 (2%) | 0.330 |
| Weakness of respiratory muscles, n (%) | 17 (4%) | 7 (3%) | 0.632 |
| Hypoventilation, n (%) | 2 (0%) | 0 (0%) | 0.552 |
| Hypercapnic coma, n (%) | 3 (1%) | 2 (1%) | 0.765 |
| Septic shock, n (%) | 4 (1%) | 1 (0%) | 0.668 |
| Cardiogenic shock, n (%) | 0 (0%) | 2 (1%) | 0.113 |
| Hemorrhage, n (%) | 3 (1%) | 0 (0%) | 0.555 |
| Neurologic event, n (%) | 11 (3%) | 3 (1%) | 0.326 |
| Surgery, n (%) | 3 (1%) | 1 (0%) | 0.999 |

*A same patient may have several criteria or reasons for reintubation**.**
